# Global Problem of Hospital Detention Practices

**DOI:** 10.15171/ijhpm.2020.10

**Published:** 2020-02-02

**Authors:** Krisna Handayani, Tyas C. Sijbranda, Maurits A. Westenberg, Nuria Rossell, Mei N. Sitaresmi, Gertjan JL Kaspers, Saskia Mostert

**Affiliations:** ^1^Faculty of Medicine, Universitas Gadjah Mada, Dr. Sardjito Hospital, Yogyakarta, Indonesia.; ^2^Amsterdam University Medical Center, VU University, Amsterdam, The Netherlands.; ^3^Amsterdam Institute for Social Science Research, University of Amsterdam, Amsterdam, The Netherlands.; ^4^Department of Oncology, Benjamin Bloom Hospital, San Salvador, El Salvador.; ^5^Princess Máxima Center for Pediatric Oncology, Utrecht, The Netherlands.

**Keywords:** Hospital Detention Practices, Corruption, Universal Health Coverage

## Abstract

Although an official definition by the World Health Organization (WHO) or any other authority is currently lacking, hospital detention practices (HDP) can be described as: "refusing release of either living patients after medical discharge is clinically indicated or refusing release of bodies of deceased patients if families are unable to pay their hospital bills." Reports of HDP are very scarce and lack consistent terminology. Consequently, the problem’s scale is unknown. This study aimed to find evidence of HDP worldwide, explore characteristics of HDP reports, and compare countries with or without reports. PubMed and Google were examined for relevant English, Spanish, and French publications up to January 2019. Of 195 countries, HDP reports were found in 46 countries (24%) in Africa, Asia, South-America, Europe, and North-America. Most reports were published by journalists in newspapers. In most countries reports concern living adults and children who are imprisoned in public hospitals. A majority (52%) of reports were of individuals detained for at least a month. Almost all countries, with or without HDP reports, have signed the Universal Declaration of Human Rights. Countries with reported HDP have larger population size (*P* <.001), worse Corruption Perception Index score (*P*=.025), higher out-of-pocket expenditure (*P* =.024), lower Universal Health Coverage Index score (*P* =.015), and worse Press Freedom Index score (*P* =.012). We conclude that HDP are more widespread than currently acknowledged. Urgent intervention by stakeholders is required to stop HDP.

## Hospital Detention Practices

Although an official definition by the World Health Organization (WHO) or any other authority is currently lacking, hospital detention practices (HDP) can be described as: “refusing release of either living patients after medical discharge is clinically indicated or refusing release of bodies of deceased patients if families are unable to pay their hospital bills.”^1^ It concerns children and adults with both acute conditions (fi, victims of road accidents, mothers with delivery complications) or chronic diseases (fi, patients with cancer, HIV/AIDS).^1,2^ Patients can be detained inside hospitals for months receiving substandard medical care. HDP accentuate hospital overcrowding and infection risk. Sometimes patients are deserted inside hospitals by their families, and unclaimed corpses discarded in anonymous graves.^2-7^

Detention of patients infringe numerous international human rights.^2,8-12^ The Universal Declaration of Human Rights consists of 30 articles which are elaborated in 9 core international human rights treaties.^8,11^ HDP violate, for instance, the International Covenant on Civil and Political Rights which state that no person shall be detained arbitrarily, every person has the right to liberty and security of person, no person shall be detained for non-payment of a debt, and that no person shall be imprisoned under unworthy inhumane conditions like crowded places with scarce food.^2,6,8,12^ HDP also violate the International Covenant on Economic, Social and Cultural Rights which acknowledges the right of every person to social security, including social insurance, to protect people who cannot pay for required medical treatment.^2,6,8,12^ In addition, the Convention on the Rights of the Child is violated, which states that no child shall be separated from parents against their will, and that no child can be withheld from healthcare services, especially to reduce child mortality.^2,6,8,12^ The United Nations (UN) have installed both treaty-based as well as charter-based bodies that should monitor human rights compliance and violations by its member states. All 9 core international human rights treaties have a special committee of independent experts that should check its implementation (treaty-based bodies). Special Rapporteurs or Working Groups are appointed to address either specific country situations or thematic issues worldwide (charter-based bodies).^11^ Despite the clear violation of human rights involved, the UN has been reluctant to seriously address HDP.^2,5,6,8^

Reports of HDP are very scarce, particularly in medical science.^9^ Because HDP are often illegal and denied by governments, official figures are absent. Without prevalence studies, most accounts derive from anecdotal or single hospital reports.^10^ In addition, reports lack consistent terminology to allow systematic review.^1^ Therefore the extent and impact of HDP is unkown, but may be more widespread than currently reported.^1,9,10^

## Mini Review

The aims of this study are to find evidence of HDP worldwide, to explore characteristics of HDP reports, and to compare countries with or without HDP reports.

In this restropective descriptive, exploratory study the search for evidence of HDP was conducted in three languages: English, Spanish and French. We searched PubMed and Google for relevant publications up to January 31, 2019 with the search terms: (“hospital detention” and “patient hostage”) OR (“retención en hospital” and “secuestro de paciente”) OR (“emprisonnement à l’hôpital” and “patient en otage”) AND (“name of country” or “continent”).

The following characteristics of HDP reports were investigated per country: source of evidence, language of report, used terminology, number of reports, year of most recent report, age of detained patients, type of hospital, condition of patients, and duration of detention.

All countries over the world were included and divided into two groups: those with or without evidence of HDP. The following characteristics of countries were compared: signed Universal Declaration of Human Rights,^11^ ratified UN Convention on the Rights of the Child,^12^ World Bank Country Classification,^13^ population size,^14^ Corruption Perception Index,^15^ current health expenditure,^16^ out-of-pocket expenditure,^17,18^ Universal Health Coverage Index,^18-22^ and Press Freedom Index.^23^

Frequency distribution and median were computed using SPSS version 22. Comparison of characteristics between countries with or without reports of HDP were analyzed using chi-square and Mann-Whitney U test. A two-sided *P* value of .05 or less was defined as statistically significant.

## Evidence of Hospital Detention Practices Worldwide

Of 195 countries,^11^ reports of HDP have been found in 46 countries (24%). Figure and Table 1 show evidence of HDP per continent.^2,4,6,7,24-120^ HDP have most frequently been reported in Africa, Asia and South-America. Yet, accounts come from the European and North-American continent as well.

**Figure:**
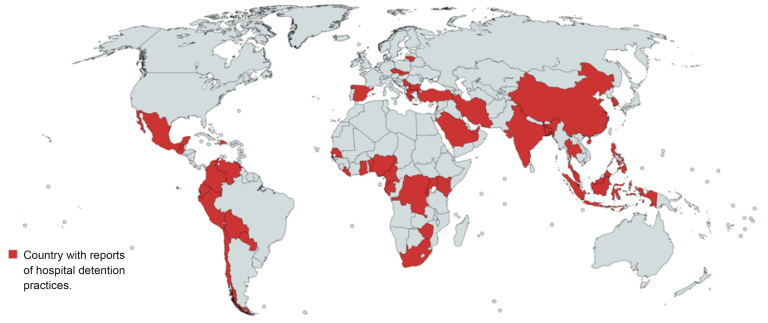
Global Map of Reported HDP. Abbreviation: HDP, Hospital Detention Practices.

**Table 1:** Reports of HDP Per Continent

**Continent**	**Countries With HDP Reports**		Names of Countries With HDP Reports*
	**n/Total n**	**(%)**	
South-America	7/12 countries	(58%)	Bolivia^#,24-28^ Chile,^29^ Colombia,^30-32^ Ecuador,^33^ Paraguay,^34^ Peru,^35,36^ Venezuela^4^
Africa	15/54 countries	(28%)	Benin,^9,37-39^ Burundi^#^,^2,3,10,40,41^ Cameroon^#^,^42-46^ Democratic Republic of Congo^#^,^28,46-49^ Equatorial Guinea,^50^ Gabon,^51^ Ghana^#^,^10,46,52-54^ Kenya^#^,^55-59^ Liberia,^3,44,60,61^ Nigeria^#^,^46,62-65^ Rwanda,^66,67^ Senegal,^68^ South Africa,^9,69^ Uganda^#^,^3,46,70-72^ Zimbabwe^3,46,73-75^
Asia	13/48 countries	(27%)	Bangladesh,^76,77^ China,^28,78-80^ India^#^,^81-85^ Indonesia^#^,^46,86-89^ Iran,^28,90^ Lebanon,^91,92^Malaysia,^28,93^ Phillippines^#^,^93-97^ Saudi-Arabia,^98,99^ South-Korea,^100^Thailand,^28,101,102^Turkey,^103,104^ United Arab Emirates^105^
Europe	8/44 countries	(18%)	Bulgaria,^27,28,106^ Czech Republic,^27^ Greece,^107-109^ Lithuania,^28^ Macedonia,^110^ Serbia,^111^ Slovakia,^27^Spain^112^
North-America	3/23 countries	(13%)	Dominican Republic,^113,114^ Guatemala,^111,115^Mexico^#116-120^
Australia and Oceania	0/14 countries	(0%)	-
Total worldwide	46/195 countries	(24%)	

Abbreviation: HDP, hospital detention practices. * Maximum of 5 references are included per country; ^#^Countries with more than 5 HDP reports.

## Characteristics of Hospital Detention Practices Reports

Of 46 countries with HDP evidence, reports were in: English (50%), both English and French (26%), Spanish (17%), both English and Spanish (4%), French (2%). Table 2 presents characteristics of HDP reports. Most reports were published by journalists in newspapers. Although scientific papers reported HDP in 14 countries, it is noteworthy that presentation of actual research data on HDP came from only 2 countries (Burundi and Kenya). The other 12 country reports in scientific papers concerned commentaries and no research articles. In most countries HDP reports concern living adults and children who are imprisoned in public hospitals for months. The used terminology in HDP reports has a wide range, including: imprisonment of impoverished patients, hospital debtor prisons, detention of insolvent patients, hospital hostage,deprivation of liberty, hostage corpse, and kidnapping.

**Table 2:** Characteristics of HDP Reports Per Country (n = 46)

**Characteristics of HDP Reports**	**Number Per 46 Countries** **No. (%)**
Source of evidence^a^	
Newspaper article	41 (89)
Scientific paper	14 (30)
Global health report	14 (30)
Human rights report	7 (15)
Personal online report	5 (11)
Documentary film	1 (2)
Private foundation report	1 (2)
Number of reports	
1-3 reports	28 (61)
>3 reports	18 (39)
Year of most recent report	
≤5 years ago	38 (83)
6-10 years ago^b^	4 (9)
>10 years ago^c^	4 (9)
Age of detained patients	
Adult and child patients	21 (46)
Adult patients	17 (37)
Child patients	8 (17)
Type of hospital	
Public hospital	19 (41)
Public and private hospital	8 (17)
Private hospital	6 (13)
Not specified	13 (28)
Condition of patients	
Alive patients	31 (67)
Alive and deceased patients	13 (28)
Deceased patients	2 (4)
Duration of detention^d^	
Months (>31 days)	24 (52)
Days (1-7 days)	9 (19)
Weeks (8-31 days)	2 (4)
Not specified	11 (24)

Abbreviation: HDP, hospital detention practices. ^a^ One country can have various sources of evidence. ^b^ Senegal, South-Korea, Colombia, Macedonia. ^c^ Chile, Ecuador, Venezuela, Serbia. ^d^ Note that reported duration of detention is skewed as newspaper articles are less likely to report on individuals detained for short periods, and if individuals are still detained the reported duration is actually the minimum period of detention.

## Comparison of CountriesWithor Without HDP Reports

Table 3 compares characteristics between countries with or without reports of HDP.^8-17^ Almost all countries, with or without HDP reports, have signed the Universal Declaration of Human Rights^8^ (respectively 100% versus 99% of countries), and ratified the UN Convention on the Rights of the Child^9^ (respectively 98% versus 97% of countries). Countries with reported HDP have a larger population size^11^ (*P* < .001), worse Corruption Perception Index score^12^ (*P* = .025), higher out-of-pocket expenditure^14,15^ (*P* = .024), lower Universal Health Coverage Index^15,16^ (*P* = .015), and worse Press Freedom Index^17^ (*P* = .012).

**Table 3:** Comparison of Characteristics Between Countries With or Without Reports of HDP

**Country Characteristics**	**Countries With HDP Reports**	**Countries Without HDP Reports**	***P***
	**(n = 46)**	**(n = 149)**	
World Bank Country Classification			
Low and middle-income countries	27%	73%	.071^a^
High-income countries	15%	85%	
Population size^c^	21 392 076 (48 450 899: 10 674 723-54 841 552)	5 615 533 (18 672 924: 1 219 878-19 676 426)	<.001^b^
Corruption Perception Index <50	82%	65%	.025^a^
Current health expenditure (% of GDP)^c^	6.0 (4.0: 4.0-8.0)	6.0 (4.0: 5.0-9.0)	.164^b^
Out-of-pocket expenditure (% of current health expenditure)			.024^b^
<15	9%	27%	
15-30	30%	28%	
30-50	43%	31%	
50-60	7%	9%	
>60	11%	5%	
Universal Health Coverage Index ≥80%	2%	16%	.015^a^
Press Freedom Index			
Very bad	11%	12%	.012^a^
Bad	46%	23%	
Problematic	28%	35%	
Fairly good	15%	18%	
Good	0%	12%	

Abbreviation: HDP: hospital detention practices. ^a^ Chi-square test; ^b^ Mann-Whitney U test; ^c^ Median (interquartile range: lower bounds 25%-upper bounds 75%).

## Discourse

This study illustrates that the problem of HDP is more widespread than currently acknowledged by policy-makers, UN, WHO, international financial institutions, health organizations, and donor countries.^1-3,5^ Reports of HDP stem from 46 countries in Africa, Asia, South-America, Europe, and North-America. In most countries HDP reports concern living adults and children who are imprisoned in public hospitals for months. Countries with reported HDP have worse corruption, higher out-of-pocket expenses, and lower universal health coverage.

Evidence of HDP is hard to find.^1,9,10^ Reports were more often published by journalists in newspapers than by scientists in medical, legal or human rights literature. Countries with HDP reports had worse press freedom. This is of course no surprise as press freedom reflects the degree of pluralism, media independence, (self)censorship and transparency that journalists, scientists and citizens have to address and correct sensitive issues regarding government performance, such as HDP.^23^

It is disturbing that almost all countries with HDP reports have signed the Universal Declaration of Human Rights and ratified the UN Convention of the Rights of the Child.^11,12^ Although HDP unequivocally violate numerous international human rights, the problem has received little or no attention from the UN. Neither from its committees of experts that should monitor the implementation of signed treaties, nor from its Special Rapporteurs and Working Groups.^4,8,9^ Condemnation of HDP by this important institute however is imperative. Their treaty-based and charter-based monitoring bodies must start to investigate and report on HDP.

HDP have numerous negative consequences. Among the general public, fear of detention may forestall or postpone seeking of conventional healthcare, and stimulate patients to attend traditional healers. This may lead to progressive or relapsed disease and unnecessary death.^1,2,6,9^ Hospital detention has a major psychological impact and is described by its victims as a traumatic experience. The financial effect of HDP is also profound. Hospital bills, that may accumulate with every extra day in detention, put significant economic pressure on the patient and their relatives.^1,2,6^ Commonly possessions that are important for their livelihood need to be sold, such as land, cattle or harvest. This pushes households deeper into longterm poverty.^5,7^

Our study underlines that HDP is associated with corruption, governmental mismanagement, dysfunctional healthcare system structures, and inadequate financial protection from health costs.^5,6^ Table 3 shows that countries with HDP reports are troubled with worse corruption.^15^ Weak state leadership in these countries with often large populations results in bad government performance. The subsequent higher out-of-pocket expenses in the health sector particularly inflict financial hardship for the poor. Health inequity remains when poor populations have limited access to medical care and risk to be detained inside hospitals if they cannot pay hospital bills.^1-3,5,6^

We found that countries with HDP reports have lower levels of health coverage. Implementation of universal health insurance significantly improves a population’s access to medical services and health outcomes.^7,20-22^ It precludes patients to be pushed into prolonged poverty while paying healthcare out of their pockets.^7,20-22^ And it also prevents HDP.^5^ Universal health coverage hereby stimulates durable development, poverty reduction, economic growth and declines socio-economic inequalty.^20-22^

This study was subject to several limitations beyond those already described. Only countries with English, Spanish or French reports of HDP were included, which implies that the 46 country accounts are likely an underrepresentation of the magnitude of the problem. HDP may occur in more countries, but are either solely reported in other languages or not reported at all. No country report therefore does not mean no presence of HDP. Further research into the prevalence of HDP across and within countries is required.

Governmental healthcare policies in various continents of the world must change rigorously to stop HDP. More critical awareness of this problem is required among policy-makers. The WHO should start by providing an official definition of the phenomenon. Both the UN and WHO must publicly condemn HDP.^1,2,6^ The UN should erect a global monitoring framework to examine HDP in its member states. The WHO should publish health recommendations advising national governments not to detain patients inside their hospitals. Corruption in the public health sector needs to be addressed.^5^ Implementation of universal healthcare coverage should be prioritized in low- and middle-income countries.^6,7,20,21^ International financial institutions, health organizations, and donor countries need to enforce that aid is employed to end HDP and achieve universal health insurance.^1,5,6^ National governments must release all imprisoned patients, stop HDP, implement legislation to make HDP actionable by law, and realize universal health coverage.^1,2,6^ These arrangements will ameliorate equal accession to medical care for patients all over the world.

## Acknowledgements

We are grateful for the support received from the Noord-Zuid Programma and AFAS Foundation.

## Ethical issues

Not applicable.

## Competing interests

Authors declare that they have no competing interests.

## Authors’ contributions

MNS, GJK, and SM conceptualized and designed the study. KH, TCS, MAW, and SM conceptualized and designed the data collection instruments. SM coordinated and supervised the data collection. KH, TCS, MAW, NR, and SM collected the data. KH and SM analyzed the data. All authors interpreted the data. KH drafted the initial manuscript. TCS, MAW, NR, MNS, GJK, and SM critically reviewed and revised the article. All authors approved the final manuscript as submitted.

## Authors’ affiliations

^1^Faculty of Medicine, Universitas Gadjah Mada, Dr. Sardjito Hospital, Yogyakarta, Indonesia. ^2^Amsterdam University Medical Center, VU University, Amsterdam, The Netherlands. ^3^Amsterdam Institute for Social Science Research, University of Amsterdam, Amsterdam, The Netherlands. ^4^Department of Oncology, Benjamin Bloom Hospital, San Salvador, El Salvador. ^5^Princess Máxima Center for Pediatric Oncology, Utrecht, The Netherlands.
